# Reply to Taylor et al. Comment on “Manna et al. SARS-CoV-2 Inactivation in Aerosol by Means of Radiated Microwaves. *Viruses* 2023, *15*, 1443”

**DOI:** 10.3390/v15102111

**Published:** 2023-10-18

**Authors:** Antonio Manna, Davide De Forni, Marco Bartocci, Nicola Pasculli, Barbara Poddesu, Florigio Lista, Riccardo De Santis, Donatella Amatore, Giorgia Grilli, Filippo Molinari, Alberto Sangiovanni Vincentelli, Franco Lori

**Affiliations:** 1 Elettronica S.p.A., Via Tiburtina Valeria, Km 13.700, 00131 Rome, Italy; 2 ViroStatics S.r.l., Viale Umberto I, 46, 07100 Sassari, Italy; 3 Defense Institute for Biomedical Sciences, 00184 Rome, Italy; 4 Department of EECS, University of California, Berkeley, CA 94720, USA

**Keywords:** SARS-CoV-2, SRET, COVID-19, airborne pathogens, aerosol transmission, microwave inactivation

## Abstract

SARS-CoV-2 is inactivated in aerosol (its primary mode of transmission) by means of radiated microwaves at frequencies that have been experimentally determined. Such frequencies are best predicted by the mathematical model suggested by Taylor, Margueritat and Saviot. The alignment between such mathematical prediction and the outcomes of our experiments serves to reinforce the efficacy of the radiated microwave technology and its promise in mitigating the transmission of SARS-CoV-2 in its naturally airborne state.

First of all, we thank Taylor, Margueritat and Saviot for their comments [1] that are correct in underlining that the approximate models used in our original manuscript are more appropriate for viruses with a rod-shaped structure [2]. In this respect, we wish to acknowledge that the equations and the narrative we have used came from the following reference from Barbora and Minnes [3] that was present in all preliminary versions of the manuscript but that was erroneously omitted in its final version.

The initial assumptions (i.e., the spherical shape of the virions, the homogeneous constituent medium, the VL and VT values considered similar to those measured for STMV [4]) make the model proposed by Taylor, Margueritat and Saviot most appropriate.

We further agree that solving the eigenvalue equation numerically offers a more effective method, since it provides the frequency of the dipolar mode, that is, the information needed to design inactivation experiments. 

In a fortunate coincidence, an error in the equation solving step for our calculations yielded an estimate of the resonant frequencies that were close to the ones estimated by the numerical approach proposed by Taylor, Margueritat and Saviot. Given the various approximations used in developing the models (e.g., the sphericity of particles, the homogeneity of the constituent medium, the estimations of VL and VT, i.e., the velocity of propagation of acoustic waves in viral particles, based on measurements taken on another family of viruses), we want to stress that the frequencies found with the models should be regarded as guidelines for an extended test procedure, where a range of frequencies are applied, to make sure that the correct ones are used in the apparatus. This coincidence boosted our confidence in the model, clouding a thorough re-evaluation to spot potential errors.

It is, however, reassuring how the Taylor, Margueritat and Saviot model yields frequency predictions that closely align with the observed inactivation frequencies of SARS-CoV-2 in our experimental findings, which range from 7.4 to 17.2 GHz in their prediction, and 6.5 to 17 GHz in our experiments. This not only reinforces the credibility of their model but also provides us an additional support to our results, knowing that their accurate predictions correlate with our actual experimental outcome. This further validates the effectiveness of our radiated microwaves technology, and its potential to address SARS-CoV-2 transmission in its natural airborne form.

## Data Availability

Data is contained within the article.
